# [Bis­(2-pyrid­yl-κ*N*)amine]chlorido(η^6^-hexa­methyl­benzene)­ruthenium(II) hexa­fluorido­phosphate dichloro­methane solvate

**DOI:** 10.1107/S1600536811011640

**Published:** 2011-04-07

**Authors:** Gajendra Gupta, Bruno Therrien, Jinkwon Kim

**Affiliations:** a Department of Chemistry, Kongju National University, 182 Shinkwan, Kongju, Chungnam 314-701, Republic of Korea; b Service Analytique Facultaire, Université de Neuchâtel, Ave de Bellevaux 51, CH-2000 Neuchâtel, Switzerland

## Abstract

In the title half-sandwich complex, [RuCl(η^6^-C_12_H_18_)(C_10_H_9_N_3_)]PF_6_·CH_2_Cl_2_, the ruthenium(II) ion is four-coordinated by a chloro, a hexa­methyl­benzene and a bidentate *N,N′*-chelating di(pyridin-2-yl)amine ligand. In the crystal, the amino N—H group forms a hydrogen bond with the chloro ligand of a neighbouring complex, thus forming chains along the *b* axis. Weak inter­molecular C—H⋯F and C—H⋯ Cl contacts are also observed.

## Related literature

For related structures with the same *N,N′*-chelating ligand coordinated to arene ruthenium moieties, see: Romain *et al.* (2010 ▶); Gupta *et al.* (2011 ▶); Singh *et al.* (2004 ▶). For the synthesis, see: Romain *et al.* (2010 ▶); Gupta *et al.* (2010 ▶).
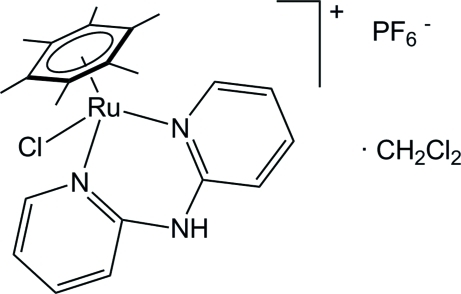


## Experimental

### 

#### Crystal data

[RuCl(C_12_H_18_)(C_10_H_9_N_3_)]PF_6_·CH_2_Cl_2_
*M* *_r_* = 699.88
Monoclinic, 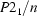

*a* = 15.5241 (4) Å
*b* = 9.1644 (2) Å
*c* = 18.9108 (5) Å
β = 93.621 (1)°
*V* = 2685.05 (12) Å^3^
*Z* = 4
Mo *K*α radiation
μ = 1.00 mm^−1^
*T* = 173 K
0.20 × 0.17 × 0.16 mm

#### Data collection

Bruker SMART CCD diffractometer
Absorption correction: multi-scan (*SADABS*; Sheldrick, 1996 ▶) *T*_min_ = 0.738, *T*_max_ = 0.871
31923 measured reflections
9327 independent reflections
7812 reflections with *I* > 2σ(*I*)
*R*_int_ = 0.030

#### Refinement

*R*[*F* ^2^ > 2σ(*F* ^2^)] = 0.033
*wR*(*F* ^2^) = 0.087
*S* = 1.06
9327 reflections
344 parameters
H atoms treated by a mixture of independent and constrained refinement
Δρ_max_ = 0.77 e Å^−3^
Δρ_min_ = −0.78 e Å^−3^

### 

Data collection: *SMART* (Bruker, 1999 ▶); cell refinement: *SMART* and *SAINT* (Bruker, 1999 ▶); data reduction: *SAINT*; program(s) used to solve structure: *SIR97* (Altomare *et al.*, 1999 ▶); program(s) used to refine structure: *SHELXTL* (Sheldrick, 2008 ▶); molecular graphics: *SHELXTL*; software used to prepare material for publication: *SHELXTL*.

## Supplementary Material

Crystal structure: contains datablocks I, global. DOI: 10.1107/S1600536811011640/om2411sup1.cif

Structure factors: contains datablocks I. DOI: 10.1107/S1600536811011640/om2411Isup2.hkl

Additional supplementary materials:  crystallographic information; 3D view; checkCIF report

## Figures and Tables

**Table 1:** Hydrogen-bond geometry (Å, °)

*D*—H⋯*A*	*D*—H	H⋯*A*	*D*⋯*A*	*D*—H⋯*A*
N2—H2*A*⋯Cl1^i^	0.85 (3)	2.51 (3)	3.3493 (17)	170 (3)
C3—H3⋯F3^ii^	0.95	2.52	3.351 (3)	146
C8—H8⋯F5^i^	0.95	2.55	3.435 (3)	155
C20—H20*A*⋯Cl3^iii^	0.98	2.78	3.626 (2)	144
C20—H20*B*⋯F2^iv^	0.98	2.54	3.424 (3)	151

Symmetry codes: (i) 
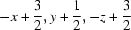
; (ii) 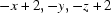; (iii) 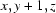; (iv) 
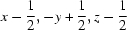
.

**Fig. 1.**
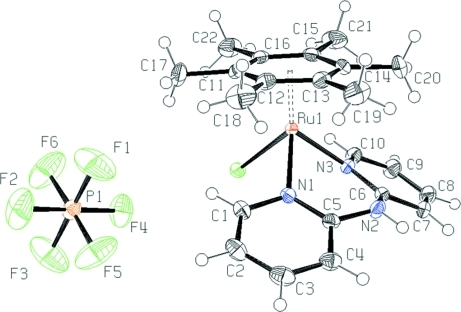
The molecular structure of [RuCl(η2-C10H9N3-κ2-N,N')(η6-C12H18)]PF6. Displacement ellipsoids are drawn at the 50% probability level.

## supplementary crystallographic information

### Comment

The title complex shows typical three legged piano-stool geometry with the ruthenium(II) atom being coordinated by a hexamethylbenzene ligand occupying one face of the octahedron, a terminal chloro and a bidentate *N,N'*-chelating ligand, see Fig. 1. The di(pyridin-2-yl)amine ligand acts as a bidentate chelating ligand through its two pyridyl groups, and the Ru-N distances are essentially equivalent at 2.099 (2) and 2.098 (2) Å. The aromatic ring of the hexamethylbenzene is planar and the Ru-centroid distance is 1.690 (2) Å. The Ru-Cl distance is 2.4108 (4) Å, similar to that found in other chloro arene ruthenium complexes (Singh *et al.*, 2004; Gupta *et al.*, 2011).

In the crystal packing, the N-H amino group is involved in a H-bonded interaction with the chloro ligand of a neighbouring complex, thus forming infinite one-dimensional chains along the *b* axis. Moreover, there are weak intermolecular contacts of the type C—H···F and C—H··· Cl.

### Experimental

The synthesis of the title compound has been reported (Romain *et al.*, 2010; Gupta *et al.*, 2011). Yellow-orange crystals for X-ray diffraction analysis were obtained by slow diffusion of hexane into a dichloromethane solution of the title complex.

### Refinement

The H atoms were included in calculated positions and refined using a riding model, with C—H = 0.93–0.96 Å and with *U*_iso_(H) = 1.2 (1.5 for methyl) times *U*_eq_(C), except for the H atom of the N—H group which was found in the Fourier difference map and refined isotropically.

### Crystal data

**Table:**

[RuCl(C_12_H_18_)(C_10_H_9_N_3_)]PF_6_·CH_2_Cl_2_	*F*(000) = 1408
*M**_r_* = 699.88	*D*_x_ = 1.731 Mg m^−^^3^
Monoclinic, *P*2_1_/*n*	Mo *K*α radiation, λ = 0.71073 Å
Hall symbol: -P 2yn	Cell parameters from 550 reflections
*a* = 15.5241 (4) Å	θ = 1.7–27.0°
*b* = 9.1644 (2) Å	µ = 1.00 mm^−^^1^
*c* = 18.9108 (5) Å	*T* = 173 K
β = 93.621 (1)°	Block, orange
*V* = 2685.05 (12) Å^3^	0.20 × 0.17 × 0.16 mm
*Z* = 4	

### Data collection

**Table:**

Bruker SMART CCD diffractometer	9327 independent reflections
Radiation source: fine-focus sealed tube	7812 reflections with *I* > 2σ(*I*)
graphite	*R*_int_ = 0.030
Detector resolution: 0 pixels mm^-1^	θ_max_ = 33.9°, θ_min_ = 1.7°
ω scans	*h* = −22→24
Absorption correction: multi-scan (*SADABS*; Sheldrick, 1996)	*k* = −14→11
*T*_min_ = 0.738, *T*_max_ = 0.871	*l* = −25→29
31923 measured reflections	

### Refinement

**Table:**

Refinement on *F*^2^	Primary atom site location: structure-invariant direct methods
Least-squares matrix: full	Secondary atom site location: difference Fourier map
*R*[*F*^2^ > 2σ(*F*^2^)] = 0.033	Hydrogen site location: inferred from neighbouring sites
*wR*(*F*^2^) = 0.087	H atoms treated by a mixture of independent and constrained refinement
*S* = 1.06	*w* = 1/[σ^2^(*F*_o_^2^) + (0.0383*P*)^2^ + 2.053*P*] where *P* = (*F*_o_^2^ + 2*F*_c_^2^)/3
9327 reflections	(Δ/σ)_max_ = 0.003
344 parameters	Δρ_max_ = 0.77 e Å^−^^3^
0 restraints	Δρ_min_ = −0.78 e Å^−^^3^

### Special details

**Table:**

Geometry. All esds (except the esd in the dihedral angle between two l.s. planes) are estimated using the full covariance matrix. The cell esds are taken into account individually in the estimation of esds in distances, angles and torsion angles; correlations between esds in cell parameters are only used when they are defined by crystal symmetry. An approximate (isotropic) treatment of cell esds is used for estimating esds involving l.s. planes.
Refinement. Refinement of *F*^2^ against ALL reflections. The weighted *R*-factor *wR* and goodness of fit *S* are based on *F*^2^, conventional *R*-factors *R* are based on *F*, with *F* set to zero for negative *F*^2^. The threshold expression of *F*^2^ > σ(*F*^2^) is used only for calculating *R*-factors(gt) *etc*. and is not relevant to the choice of reflections for refinement. *R*-factors based on *F*^2^ are statistically about twice as large as those based on *F*, and *R*- factors based on ALL data will be even larger.

### Fractional atomic coordinates and isotropic or equivalent isotropic displacement parameters (Å^2^)

**Table:**

	*x*	*y*	*z*	*U*_iso_*/*U*_eq_	
Ru1	0.623752 (8)	0.316216 (13)	0.871467 (6)	0.01424 (4)	
Cl1	0.62147 (3)	0.05324 (4)	0.86885 (2)	0.02066 (8)	
N1	0.75701 (10)	0.29849 (15)	0.86021 (8)	0.0181 (3)	
N2	0.74819 (10)	0.42939 (17)	0.75231 (8)	0.0222 (3)	
H2A	0.7794 (18)	0.472 (3)	0.7233 (15)	0.038 (7)*	
N3	0.61569 (10)	0.30532 (15)	0.76042 (8)	0.0176 (3)	
C1	0.80663 (12)	0.2324 (2)	0.91254 (10)	0.0237 (4)	
H1	0.7790	0.1776	0.9472	0.028*	
C2	0.89516 (13)	0.2416 (2)	0.91737 (12)	0.0306 (4)	
H2	0.9280	0.1945	0.9548	0.037*	
C3	0.93565 (14)	0.3209 (2)	0.86646 (13)	0.0323 (5)	
H3	0.9967	0.3312	0.8694	0.039*	
C4	0.88661 (13)	0.3843 (2)	0.81186 (11)	0.0265 (4)	
H4	0.9133	0.4383	0.7764	0.032*	
C5	0.79686 (12)	0.36842 (19)	0.80912 (9)	0.0196 (3)	
C6	0.67229 (11)	0.37280 (19)	0.72068 (9)	0.0183 (3)	
C7	0.65632 (13)	0.3905 (2)	0.64760 (9)	0.0238 (4)	
H7	0.6973	0.4385	0.6205	0.029*	
C8	0.58096 (14)	0.3377 (2)	0.61534 (10)	0.0300 (4)	
H8	0.5682	0.3520	0.5660	0.036*	
C9	0.52350 (14)	0.2629 (3)	0.65590 (10)	0.0285 (4)	
H9	0.4716	0.2231	0.6347	0.034*	
C10	0.54359 (12)	0.2481 (2)	0.72714 (9)	0.0226 (3)	
H10	0.5051	0.1950	0.7546	0.027*	
C11	0.59093 (13)	0.33487 (19)	0.98353 (9)	0.0215 (3)	
C12	0.64864 (13)	0.4479 (2)	0.96903 (10)	0.0238 (4)	
C13	0.63113 (13)	0.54226 (19)	0.90926 (10)	0.0244 (4)	
C14	0.55431 (14)	0.5232 (2)	0.86567 (10)	0.0253 (4)	
C15	0.49318 (12)	0.4121 (2)	0.88188 (10)	0.0234 (4)	
C16	0.51229 (12)	0.31772 (19)	0.93991 (10)	0.0210 (3)	
C17	0.61053 (17)	0.2298 (3)	1.04399 (11)	0.0362 (5)	
H17A	0.6643	0.2589	1.0702	0.054*	
H17B	0.6168	0.1310	1.0251	0.054*	
H17C	0.5632	0.2314	1.0759	0.054*	
C18	0.73129 (16)	0.4720 (3)	1.01410 (13)	0.0425 (6)	
H18A	0.7244	0.5561	1.0453	0.064*	
H18B	0.7786	0.4907	0.9834	0.064*	
H18C	0.7445	0.3850	1.0429	0.064*	
C19	0.69407 (19)	0.6613 (2)	0.89334 (15)	0.0425 (6)	
H19A	0.6916	0.6792	0.8422	0.064*	
H19B	0.7526	0.6311	0.9096	0.064*	
H19C	0.6789	0.7509	0.9180	0.064*	
C20	0.5372 (2)	0.6196 (3)	0.80187 (13)	0.0474 (7)	
H20A	0.5179	0.7158	0.8171	0.071*	
H20B	0.4923	0.5755	0.7700	0.071*	
H20C	0.5903	0.6304	0.7770	0.071*	
C21	0.40948 (15)	0.3991 (3)	0.83713 (13)	0.0415 (6)	
H21A	0.3842	0.3026	0.8441	0.062*	
H21B	0.4207	0.4116	0.7871	0.062*	
H21C	0.3693	0.4747	0.8511	0.062*	
C22	0.45135 (16)	0.1958 (2)	0.95564 (14)	0.0362 (5)	
H22A	0.4105	0.1804	0.9146	0.054*	
H22B	0.4196	0.2215	0.9970	0.054*	
H22C	0.4843	0.1060	0.9655	0.054*	
P1	0.83358 (3)	−0.08437 (6)	1.08848 (3)	0.02634 (11)	
F1	0.80510 (16)	0.0740 (2)	1.10653 (13)	0.0875 (7)	
F2	0.88637 (13)	−0.0904 (3)	1.16249 (9)	0.0800 (7)	
F3	0.86296 (14)	−0.2456 (2)	1.06967 (14)	0.0859 (7)	
F4	0.78098 (12)	−0.0793 (2)	1.01404 (8)	0.0590 (5)	
F5	0.91667 (13)	−0.0269 (3)	1.05393 (10)	0.0792 (7)	
F6	0.75213 (14)	−0.1494 (3)	1.12304 (11)	0.0860 (8)	
Cl2	0.29778 (4)	0.13851 (7)	0.69513 (3)	0.04174 (14)	
Cl3	0.40491 (4)	−0.05472 (7)	0.78424 (4)	0.04606 (15)	
C23	0.30396 (15)	−0.0338 (3)	0.73642 (13)	0.0350 (5)	
H23A	0.2966	−0.1114	0.7001	0.042*	
H23B	0.2569	−0.0435	0.7691	0.042*	

### Atomic displacement parameters (Å^2^)

**Table:**

	*U*^11^	*U*^22^	*U*^33^	*U*^12^	*U*^13^	*U*^23^
Ru1	0.01582 (7)	0.01321 (6)	0.01390 (6)	0.00102 (4)	0.00260 (5)	0.00107 (4)
Cl1	0.0245 (2)	0.01458 (17)	0.02333 (19)	0.00161 (14)	0.00529 (16)	0.00003 (13)
N1	0.0172 (7)	0.0176 (6)	0.0197 (6)	0.0012 (5)	0.0017 (5)	0.0011 (5)
N2	0.0209 (7)	0.0253 (7)	0.0210 (7)	−0.0042 (6)	0.0053 (6)	0.0051 (6)
N3	0.0180 (7)	0.0189 (6)	0.0162 (6)	0.0002 (5)	0.0032 (5)	0.0019 (5)
C1	0.0233 (9)	0.0225 (8)	0.0251 (8)	0.0035 (7)	−0.0018 (7)	0.0021 (7)
C2	0.0235 (10)	0.0298 (10)	0.0372 (10)	0.0042 (8)	−0.0076 (8)	0.0009 (8)
C3	0.0170 (9)	0.0362 (11)	0.0432 (12)	0.0006 (8)	−0.0009 (8)	−0.0040 (9)
C4	0.0196 (9)	0.0266 (9)	0.0337 (10)	−0.0027 (7)	0.0050 (7)	−0.0028 (7)
C5	0.0192 (8)	0.0180 (7)	0.0218 (8)	0.0011 (6)	0.0033 (6)	−0.0022 (6)
C6	0.0194 (8)	0.0169 (7)	0.0188 (7)	0.0028 (6)	0.0039 (6)	0.0025 (6)
C7	0.0273 (9)	0.0263 (8)	0.0184 (7)	0.0046 (7)	0.0054 (7)	0.0071 (6)
C8	0.0308 (11)	0.0406 (11)	0.0183 (8)	0.0056 (9)	−0.0004 (7)	0.0055 (7)
C9	0.0262 (10)	0.0377 (11)	0.0211 (8)	−0.0003 (8)	−0.0031 (7)	0.0007 (8)
C10	0.0212 (8)	0.0259 (9)	0.0208 (8)	−0.0015 (7)	0.0017 (7)	0.0008 (6)
C11	0.0304 (10)	0.0204 (8)	0.0143 (7)	0.0036 (7)	0.0063 (7)	−0.0001 (6)
C12	0.0269 (9)	0.0238 (8)	0.0210 (8)	0.0001 (7)	0.0030 (7)	−0.0068 (6)
C13	0.0321 (10)	0.0142 (7)	0.0281 (9)	−0.0019 (7)	0.0114 (8)	−0.0031 (6)
C14	0.0335 (10)	0.0187 (8)	0.0246 (8)	0.0107 (7)	0.0082 (7)	0.0045 (6)
C15	0.0204 (8)	0.0261 (8)	0.0239 (8)	0.0079 (7)	0.0037 (7)	−0.0028 (7)
C16	0.0212 (8)	0.0190 (7)	0.0239 (8)	0.0018 (6)	0.0099 (7)	−0.0021 (6)
C17	0.0536 (14)	0.0342 (10)	0.0212 (9)	0.0055 (10)	0.0052 (9)	0.0088 (8)
C18	0.0361 (12)	0.0547 (15)	0.0356 (11)	−0.0057 (11)	−0.0059 (10)	−0.0177 (11)
C19	0.0544 (16)	0.0222 (10)	0.0532 (15)	−0.0135 (10)	0.0228 (12)	−0.0060 (9)
C20	0.0685 (18)	0.0369 (12)	0.0383 (12)	0.0245 (12)	0.0138 (12)	0.0198 (10)
C21	0.0233 (10)	0.0624 (16)	0.0381 (12)	0.0137 (11)	−0.0040 (9)	−0.0082 (11)
C22	0.0322 (11)	0.0302 (10)	0.0480 (13)	−0.0074 (9)	0.0171 (10)	−0.0006 (9)
P1	0.0245 (2)	0.0325 (3)	0.0217 (2)	−0.0028 (2)	−0.00065 (18)	−0.00108 (19)
F1	0.0957 (16)	0.0516 (11)	0.1124 (18)	0.0272 (11)	−0.0155 (14)	−0.0377 (12)
F2	0.0692 (13)	0.128 (2)	0.0388 (9)	−0.0203 (13)	−0.0277 (9)	0.0085 (11)
F3	0.0794 (14)	0.0444 (11)	0.129 (2)	0.0169 (10)	−0.0326 (13)	−0.0205 (12)
F4	0.0668 (11)	0.0758 (12)	0.0315 (7)	−0.0028 (9)	−0.0187 (7)	0.0029 (7)
F5	0.0597 (12)	0.1159 (18)	0.0652 (12)	−0.0452 (12)	0.0299 (9)	−0.0266 (12)
F6	0.0573 (12)	0.145 (2)	0.0575 (12)	−0.0489 (13)	0.0167 (9)	−0.0009 (13)
Cl2	0.0485 (3)	0.0372 (3)	0.0377 (3)	−0.0002 (3)	−0.0113 (2)	0.0066 (2)
Cl3	0.0356 (3)	0.0451 (3)	0.0571 (4)	0.0073 (3)	−0.0008 (3)	0.0181 (3)
C23	0.0355 (12)	0.0315 (10)	0.0377 (11)	−0.0056 (9)	−0.0006 (9)	0.0020 (9)

### Geometric parameters (Å, °)

**Table:**

Ru1—N3	2.0982 (15)	C12—C18	1.511 (3)
Ru1—N1	2.0993 (15)	C13—C14	1.417 (3)
Ru1—C14	2.1808 (18)	C13—C19	1.507 (3)
Ru1—C13	2.1920 (18)	C14—C15	1.438 (3)
Ru1—C11	2.2173 (17)	C14—C20	1.506 (3)
Ru1—C12	2.2178 (18)	C15—C16	1.414 (3)
Ru1—C16	2.2255 (19)	C15—C21	1.510 (3)
Ru1—C15	2.2292 (18)	C16—C22	1.506 (3)
Ru1—Cl1	2.4108 (4)	C17—H17A	0.9800
N1—C5	1.343 (2)	C17—H17B	0.9800
N1—C1	1.358 (2)	C17—H17C	0.9800
N2—C6	1.388 (2)	C18—H18A	0.9800
N2—C5	1.391 (2)	C18—H18B	0.9800
N2—H2A	0.85 (3)	C18—H18C	0.9800
N3—C6	1.343 (2)	C19—H19A	0.9800
N3—C10	1.355 (2)	C19—H19B	0.9800
C1—C2	1.374 (3)	C19—H19C	0.9800
C1—H1	0.9500	C20—H20A	0.9800
C2—C3	1.388 (3)	C20—H20B	0.9800
C2—H2	0.9500	C20—H20C	0.9800
C3—C4	1.373 (3)	C21—H21A	0.9800
C3—H3	0.9500	C21—H21B	0.9800
C4—C5	1.399 (3)	C21—H21C	0.9800
C4—H4	0.9500	C22—H22A	0.9800
C6—C7	1.398 (2)	C22—H22B	0.9800
C7—C8	1.373 (3)	C22—H22C	0.9800
C7—H7	0.9500	P1—F1	1.5608 (19)
C8—C9	1.393 (3)	P1—F5	1.5730 (18)
C8—H8	0.9500	P1—F6	1.5766 (19)
C9—C10	1.370 (3)	P1—F2	1.5776 (16)
C9—H9	0.9500	P1—F4	1.5830 (14)
C10—H10	0.9500	P1—F3	1.593 (2)
C11—C12	1.408 (3)	Cl2—C23	1.761 (2)
C11—C16	1.438 (3)	Cl3—C23	1.770 (2)
C11—C17	1.511 (3)	C23—H23A	0.9900
C12—C13	1.436 (3)	C23—H23B	0.9900
			
N3—Ru1—N1	83.79 (6)	C11—C12—Ru1	71.48 (10)
N3—Ru1—C14	89.60 (6)	C13—C12—Ru1	70.03 (10)
N1—Ru1—C14	123.32 (7)	C18—C12—Ru1	130.24 (15)
N3—Ru1—C13	111.71 (6)	C14—C13—C12	119.61 (17)
N1—Ru1—C13	94.28 (7)	C14—C13—C19	120.4 (2)
C14—Ru1—C13	37.81 (8)	C12—C13—C19	120.0 (2)
N3—Ru1—C11	163.23 (7)	C14—C13—Ru1	70.67 (10)
N1—Ru1—C11	112.97 (7)	C12—C13—Ru1	71.98 (10)
C14—Ru1—C11	80.69 (7)	C19—C13—Ru1	129.79 (15)
C13—Ru1—C11	68.04 (7)	C13—C14—C15	120.39 (16)
N3—Ru1—C12	148.73 (7)	C13—C14—C20	119.5 (2)
N1—Ru1—C12	90.31 (7)	C15—C14—C20	120.1 (2)
C14—Ru1—C12	68.18 (7)	C13—C14—Ru1	71.52 (10)
C13—Ru1—C12	38.00 (7)	C15—C14—Ru1	72.80 (10)
C11—Ru1—C12	37.01 (7)	C20—C14—Ru1	127.76 (15)
N3—Ru1—C16	125.66 (7)	C16—C15—C14	119.30 (17)
N1—Ru1—C16	150.10 (7)	C16—C15—C21	121.2 (2)
C14—Ru1—C16	67.90 (7)	C14—C15—C21	119.49 (19)
C13—Ru1—C16	80.27 (7)	C16—C15—Ru1	71.35 (10)
C11—Ru1—C16	37.78 (7)	C14—C15—Ru1	69.15 (10)
C12—Ru1—C16	67.28 (7)	C21—C15—Ru1	132.43 (14)
N3—Ru1—C15	96.32 (6)	C15—C16—C11	120.49 (17)
N1—Ru1—C15	161.23 (7)	C15—C16—C22	120.23 (19)
C14—Ru1—C15	38.05 (8)	C11—C16—C22	119.27 (18)
C13—Ru1—C15	68.16 (7)	C15—C16—Ru1	71.64 (11)
C11—Ru1—C15	67.67 (7)	C11—C16—Ru1	70.80 (10)
C12—Ru1—C15	79.85 (7)	C22—C16—Ru1	129.16 (13)
C16—Ru1—C15	37.01 (7)	C11—C17—H17A	109.5
N3—Ru1—Cl1	86.10 (4)	C11—C17—H17B	109.5
N1—Ru1—Cl1	86.21 (4)	H17A—C17—H17B	109.5
C14—Ru1—Cl1	149.52 (6)	C11—C17—H17C	109.5
C13—Ru1—Cl1	162.15 (5)	H17A—C17—H17C	109.5
C11—Ru1—Cl1	95.32 (5)	H17B—C17—H17C	109.5
C12—Ru1—Cl1	124.22 (5)	C12—C18—H18A	109.5
C16—Ru1—Cl1	90.41 (5)	C12—C18—H18B	109.5
C15—Ru1—Cl1	112.55 (5)	H18A—C18—H18B	109.5
C5—N1—C1	118.13 (16)	C12—C18—H18C	109.5
C5—N1—Ru1	122.54 (12)	H18A—C18—H18C	109.5
C1—N1—Ru1	118.56 (13)	H18B—C18—H18C	109.5
C6—N2—C5	126.00 (15)	C13—C19—H19A	109.5
C6—N2—H2A	113.3 (18)	C13—C19—H19B	109.5
C5—N2—H2A	112.2 (18)	H19A—C19—H19B	109.5
C6—N3—C10	117.98 (15)	C13—C19—H19C	109.5
C6—N3—Ru1	122.58 (12)	H19A—C19—H19C	109.5
C10—N3—Ru1	118.60 (12)	H19B—C19—H19C	109.5
N1—C1—C2	122.70 (19)	C14—C20—H20A	109.5
N1—C1—H1	118.6	C14—C20—H20B	109.5
C2—C1—H1	118.6	H20A—C20—H20B	109.5
C1—C2—C3	118.73 (19)	C14—C20—H20C	109.5
C1—C2—H2	120.6	H20A—C20—H20C	109.5
C3—C2—H2	120.6	H20B—C20—H20C	109.5
C4—C3—C2	119.31 (19)	C15—C21—H21A	109.5
C4—C3—H3	120.3	C15—C21—H21B	109.5
C2—C3—H3	120.3	H21A—C21—H21B	109.5
C3—C4—C5	119.19 (19)	C15—C21—H21C	109.5
C3—C4—H4	120.4	H21A—C21—H21C	109.5
C5—C4—H4	120.4	H21B—C21—H21C	109.5
N1—C5—N2	119.67 (16)	C16—C22—H22A	109.5
N1—C5—C4	121.75 (17)	C16—C22—H22B	109.5
N2—C5—C4	118.57 (17)	H22A—C22—H22B	109.5
N3—C6—N2	119.91 (15)	C16—C22—H22C	109.5
N3—C6—C7	121.68 (17)	H22A—C22—H22C	109.5
N2—C6—C7	118.40 (16)	H22B—C22—H22C	109.5
C8—C7—C6	119.44 (18)	F1—P1—F5	91.77 (14)
C8—C7—H7	120.3	F1—P1—F6	90.87 (15)
C6—C7—H7	120.3	F5—P1—F6	177.30 (15)
C7—C8—C9	119.08 (18)	F1—P1—F2	88.70 (13)
C7—C8—H8	120.5	F5—P1—F2	89.24 (12)
C9—C8—H8	120.5	F6—P1—F2	90.24 (12)
C10—C9—C8	118.44 (19)	F1—P1—F4	91.70 (11)
C10—C9—H9	120.8	F5—P1—F4	90.64 (11)
C8—C9—H9	120.8	F6—P1—F4	89.86 (11)
N3—C10—C9	123.26 (18)	F2—P1—F4	179.59 (14)
N3—C10—H10	118.4	F1—P1—F3	179.65 (17)
C9—C10—H10	118.4	F5—P1—F3	87.92 (14)
C12—C11—C16	119.76 (16)	F6—P1—F3	89.44 (15)
C12—C11—C17	121.06 (19)	F2—P1—F3	91.45 (13)
C16—C11—C17	119.18 (18)	F4—P1—F3	88.15 (11)
C12—C11—Ru1	71.52 (10)	Cl2—C23—Cl3	110.21 (12)
C16—C11—Ru1	71.42 (10)	Cl2—C23—H23A	109.6
C17—C11—Ru1	128.81 (14)	Cl3—C23—H23A	109.6
C11—C12—C13	120.36 (17)	Cl2—C23—H23B	109.6
C11—C12—C18	121.75 (19)	Cl3—C23—H23B	109.6
C13—C12—C18	117.87 (19)	H23A—C23—H23B	108.1
			
N3—Ru1—N1—C5	40.39 (14)	N1—Ru1—C13—C14	143.33 (11)
C14—Ru1—N1—C5	−45.02 (16)	C11—Ru1—C13—C14	−103.61 (12)
C13—Ru1—N1—C5	−71.01 (14)	C12—Ru1—C13—C14	−131.67 (17)
C11—Ru1—N1—C5	−138.95 (13)	C16—Ru1—C13—C14	−66.32 (11)
C12—Ru1—N1—C5	−108.84 (14)	C15—Ru1—C13—C14	−29.85 (11)
C16—Ru1—N1—C5	−148.91 (14)	Cl1—Ru1—C13—C14	−125.76 (17)
C15—Ru1—N1—C5	−51.0 (3)	N3—Ru1—C13—C12	−170.01 (11)
Cl1—Ru1—N1—C5	126.88 (14)	N1—Ru1—C13—C12	−85.01 (12)
N3—Ru1—N1—C1	−149.88 (14)	C14—Ru1—C13—C12	131.67 (17)
C14—Ru1—N1—C1	124.71 (14)	C11—Ru1—C13—C12	28.06 (11)
C13—Ru1—N1—C1	98.72 (14)	C16—Ru1—C13—C12	65.35 (12)
C11—Ru1—N1—C1	30.78 (15)	C15—Ru1—C13—C12	101.82 (12)
C12—Ru1—N1—C1	60.89 (14)	Cl1—Ru1—C13—C12	5.9 (3)
C16—Ru1—N1—C1	20.8 (2)	N3—Ru1—C13—C19	−55.6 (2)
C15—Ru1—N1—C1	118.8 (2)	N1—Ru1—C13—C19	29.4 (2)
Cl1—Ru1—N1—C1	−63.39 (13)	C14—Ru1—C13—C19	−113.9 (3)
N1—Ru1—N3—C6	−39.46 (13)	C11—Ru1—C13—C19	142.5 (2)
C14—Ru1—N3—C6	84.14 (14)	C12—Ru1—C13—C19	114.4 (3)
C13—Ru1—N3—C6	52.69 (15)	C16—Ru1—C13—C19	179.8 (2)
C11—Ru1—N3—C6	138.4 (2)	C15—Ru1—C13—C19	−143.7 (2)
C12—Ru1—N3—C6	40.8 (2)	Cl1—Ru1—C13—C19	120.3 (2)
C16—Ru1—N3—C6	146.23 (13)	C12—C13—C14—C15	1.3 (3)
C15—Ru1—N3—C6	121.66 (14)	C19—C13—C14—C15	−178.40 (18)
Cl1—Ru1—N3—C6	−126.06 (13)	Ru1—C13—C14—C15	56.12 (16)
N1—Ru1—N3—C10	151.27 (14)	C12—C13—C14—C20	−178.35 (18)
C14—Ru1—N3—C10	−85.14 (14)	C19—C13—C14—C20	1.9 (3)
C13—Ru1—N3—C10	−116.59 (14)	Ru1—C13—C14—C20	−123.56 (18)
C11—Ru1—N3—C10	−30.8 (3)	C12—C13—C14—Ru1	−54.79 (15)
C12—Ru1—N3—C10	−128.45 (15)	C19—C13—C14—Ru1	125.48 (18)
C16—Ru1—N3—C10	−23.04 (16)	N3—Ru1—C14—C13	−127.75 (11)
C15—Ru1—N3—C10	−47.61 (14)	N1—Ru1—C14—C13	−45.46 (13)
Cl1—Ru1—N3—C10	64.67 (13)	C11—Ru1—C14—C13	65.98 (11)
C5—N1—C1—C2	3.9 (3)	C12—Ru1—C14—C13	29.69 (11)
Ru1—N1—C1—C2	−166.30 (16)	C16—Ru1—C14—C13	103.05 (12)
N1—C1—C2—C3	−0.4 (3)	C15—Ru1—C14—C13	131.44 (16)
C1—C2—C3—C4	−1.8 (3)	Cl1—Ru1—C14—C13	150.63 (10)
C2—C3—C4—C5	0.5 (3)	N3—Ru1—C14—C15	100.80 (11)
C1—N1—C5—N2	175.93 (16)	N1—Ru1—C14—C15	−176.91 (10)
Ru1—N1—C5—N2	−14.3 (2)	C13—Ru1—C14—C15	−131.44 (16)
C1—N1—C5—C4	−5.2 (3)	C11—Ru1—C14—C15	−65.46 (11)
Ru1—N1—C5—C4	164.55 (14)	C12—Ru1—C14—C15	−101.75 (12)
C6—N2—C5—N1	−34.9 (3)	C16—Ru1—C14—C15	−28.40 (10)
C6—N2—C5—C4	146.20 (19)	Cl1—Ru1—C14—C15	19.19 (17)
C3—C4—C5—N1	3.1 (3)	N3—Ru1—C14—C20	−14.3 (2)
C3—C4—C5—N2	−178.03 (18)	N1—Ru1—C14—C20	68.0 (2)
C10—N3—C6—N2	−178.17 (16)	C13—Ru1—C14—C20	113.5 (3)
Ru1—N3—C6—N2	12.5 (2)	C11—Ru1—C14—C20	179.5 (2)
C10—N3—C6—C7	3.0 (3)	C12—Ru1—C14—C20	143.2 (2)
Ru1—N3—C6—C7	−166.33 (13)	C16—Ru1—C14—C20	−143.5 (2)
C5—N2—C6—N3	36.0 (3)	C15—Ru1—C14—C20	−115.1 (3)
C5—N2—C6—C7	−145.18 (18)	Cl1—Ru1—C14—C20	−95.9 (2)
N3—C6—C7—C8	0.0 (3)	C13—C14—C15—C16	−2.8 (3)
N2—C6—C7—C8	−178.84 (18)	C20—C14—C15—C16	176.85 (18)
C6—C7—C8—C9	−2.3 (3)	Ru1—C14—C15—C16	52.69 (15)
C7—C8—C9—C10	1.6 (3)	C13—C14—C15—C21	176.60 (18)
C6—N3—C10—C9	−3.8 (3)	C20—C14—C15—C21	−3.7 (3)
Ru1—N3—C10—C9	165.96 (16)	Ru1—C14—C15—C21	−127.89 (18)
C8—C9—C10—N3	1.5 (3)	C13—C14—C15—Ru1	−55.51 (15)
N3—Ru1—C11—C12	−121.3 (2)	C20—C14—C15—Ru1	124.16 (19)
N1—Ru1—C11—C12	56.45 (12)	N3—Ru1—C15—C16	145.85 (11)
C14—Ru1—C11—C12	−65.90 (12)	N1—Ru1—C15—C16	−124.88 (19)
C13—Ru1—C11—C12	−28.76 (11)	C14—Ru1—C15—C16	−132.94 (16)
C16—Ru1—C11—C12	−131.64 (16)	C13—Ru1—C15—C16	−103.26 (12)
C15—Ru1—C11—C12	−103.21 (12)	C11—Ru1—C15—C16	−28.99 (10)
Cl1—Ru1—C11—C12	144.58 (10)	C12—Ru1—C15—C16	−65.52 (11)
N3—Ru1—C11—C16	10.4 (3)	Cl1—Ru1—C15—C16	57.46 (11)
N1—Ru1—C11—C16	−171.91 (9)	N3—Ru1—C15—C14	−81.21 (11)
C14—Ru1—C11—C16	65.74 (11)	N1—Ru1—C15—C14	8.1 (2)
C13—Ru1—C11—C16	102.89 (11)	C13—Ru1—C15—C14	29.68 (11)
C12—Ru1—C11—C16	131.64 (16)	C11—Ru1—C15—C14	103.96 (12)
C15—Ru1—C11—C16	28.43 (10)	C12—Ru1—C15—C14	67.42 (11)
Cl1—Ru1—C11—C16	−83.78 (9)	C16—Ru1—C15—C14	132.94 (16)
N3—Ru1—C11—C17	123.3 (2)	Cl1—Ru1—C15—C14	−169.60 (9)
N1—Ru1—C11—C17	−59.0 (2)	N3—Ru1—C15—C21	30.2 (2)
C14—Ru1—C11—C17	178.7 (2)	N1—Ru1—C15—C21	119.5 (3)
C13—Ru1—C11—C17	−144.2 (2)	C14—Ru1—C15—C21	111.4 (3)
C12—Ru1—C11—C17	−115.4 (2)	C13—Ru1—C15—C21	141.1 (2)
C16—Ru1—C11—C17	112.9 (2)	C11—Ru1—C15—C21	−144.6 (2)
C15—Ru1—C11—C17	141.4 (2)	C12—Ru1—C15—C21	178.9 (2)
Cl1—Ru1—C11—C17	29.2 (2)	C16—Ru1—C15—C21	−115.6 (3)
C16—C11—C12—C13	−2.6 (3)	Cl1—Ru1—C15—C21	−58.2 (2)
C17—C11—C12—C13	176.89 (18)	C14—C15—C16—C11	1.6 (3)
Ru1—C11—C12—C13	52.13 (15)	C21—C15—C16—C11	−177.77 (18)
C16—C11—C12—C18	178.88 (18)	Ru1—C15—C16—C11	53.32 (15)
C17—C11—C12—C18	−1.7 (3)	C14—C15—C16—C22	−176.89 (18)
Ru1—C11—C12—C18	−126.43 (19)	C21—C15—C16—C22	3.7 (3)
C16—C11—C12—Ru1	−54.68 (15)	Ru1—C15—C16—C22	−125.22 (17)
C17—C11—C12—Ru1	124.76 (18)	C14—C15—C16—Ru1	−51.68 (15)
N3—Ru1—C12—C11	151.63 (13)	C21—C15—C16—Ru1	128.92 (18)
N1—Ru1—C12—C11	−129.88 (11)	C12—C11—C16—C15	1.0 (3)
C14—Ru1—C12—C11	103.99 (12)	C17—C11—C16—C15	−178.42 (18)
C13—Ru1—C12—C11	133.55 (17)	Ru1—C11—C16—C15	−53.70 (15)
C16—Ru1—C12—C11	29.75 (11)	C12—C11—C16—C22	179.58 (18)
C15—Ru1—C12—C11	66.19 (12)	C17—C11—C16—C22	0.1 (3)
Cl1—Ru1—C12—C11	−44.26 (13)	Ru1—C11—C16—C22	124.85 (17)
N3—Ru1—C12—C13	18.08 (19)	C12—C11—C16—Ru1	54.73 (15)
N1—Ru1—C12—C13	96.56 (12)	C17—C11—C16—Ru1	−124.72 (17)
C14—Ru1—C12—C13	−29.56 (12)	N3—Ru1—C16—C15	−43.37 (13)
C11—Ru1—C12—C13	−133.55 (17)	N1—Ru1—C16—C15	148.04 (12)
C16—Ru1—C12—C13	−103.80 (12)	C14—Ru1—C16—C15	29.14 (11)
C15—Ru1—C12—C13	−67.37 (12)	C13—Ru1—C16—C15	66.44 (12)
Cl1—Ru1—C12—C13	−177.81 (9)	C11—Ru1—C16—C15	132.97 (15)
N3—Ru1—C12—C18	−92.0 (2)	C12—Ru1—C16—C15	103.78 (12)
N1—Ru1—C12—C18	−13.6 (2)	Cl1—Ru1—C16—C15	−128.87 (10)
C14—Ru1—C12—C18	−139.7 (2)	N3—Ru1—C16—C11	−176.34 (9)
C13—Ru1—C12—C18	−110.1 (2)	N1—Ru1—C16—C11	15.07 (17)
C11—Ru1—C12—C18	116.3 (2)	C14—Ru1—C16—C11	−103.83 (11)
C16—Ru1—C12—C18	146.1 (2)	C13—Ru1—C16—C11	−66.53 (11)
C15—Ru1—C12—C18	−177.5 (2)	C12—Ru1—C16—C11	−29.19 (10)
Cl1—Ru1—C12—C18	72.1 (2)	C15—Ru1—C16—C11	−132.97 (15)
C11—C12—C13—C14	1.4 (3)	Cl1—Ru1—C16—C11	98.17 (9)
C18—C12—C13—C14	180.00 (18)	N3—Ru1—C16—C22	71.1 (2)
Ru1—C12—C13—C14	54.17 (15)	N1—Ru1—C16—C22	−97.5 (2)
C11—C12—C13—C19	−178.90 (18)	C14—Ru1—C16—C22	143.6 (2)
C18—C12—C13—C19	−0.3 (3)	C13—Ru1—C16—C22	−179.1 (2)
Ru1—C12—C13—C19	−126.10 (18)	C11—Ru1—C16—C22	−112.6 (2)
C11—C12—C13—Ru1	−52.79 (16)	C12—Ru1—C16—C22	−141.8 (2)
C18—C12—C13—Ru1	125.83 (18)	C15—Ru1—C16—C22	114.5 (2)
N3—Ru1—C13—C14	58.32 (12)	Cl1—Ru1—C16—C22	−14.42 (19)

### Hydrogen-bond geometry (Å, °)

**Table:**

*D*—H···*A*	*D*—H	H···*A*	*D*···*A*	*D*—H···*A*
N2—H2A···Cl1^i^	0.85 (3)	2.51 (3)	3.3493 (17)	170 (3)
C3—H3···F3^ii^	0.95	2.52	3.351 (3)	146
C8—H8···F5^i^	0.95	2.55	3.435 (3)	155
C20—H20A···Cl3^iii^	0.98	2.78	3.626 (2)	144
C20—H20B···F2^iv^	0.98	2.54	3.424 (3)	151

Symmetry codes: (i) −*x*+3/2, *y*+1/2, −*z*+3/2; (ii) −*x*+2, −*y*, −*z*+2; (iii) *x*, *y*+1, *z*; (iv) *x*−1/2, −*y*+1/2, *z*−1/2.
